# The Effect of Prone and Supine Limb Positioning on the Radiographic Evaluation of Posterolateral Plate Fixation of the Posterior Malleolus

**DOI:** 10.1007/s43465-023-01066-3

**Published:** 2024-01-22

**Authors:** Preemal Patel, Zoe Little, Phillip Beak, Rachel Williams, Alex Trompeter

**Affiliations:** 1 Department of Trauma and Orthopaedics, St George’s University Hospital NHS Foundation Trust, St George’s University, London, UK; 2 Trauma and Orthopaedics, Kingston Hospital, London, UK; 3 Department of Radiology, St George’s University Hospitals NHS Foundation Trust, London, UK

**Keywords:** Trauma, Ankle fractures, Radiographic study, Posterior malleolus, Posterolateral approach

## Abstract

**Aim:**

To facilitate the posterolateral approach to the posterior malleolus patients are often positioned prone initially, then turned supine to complete fixation at the medial malleolus. We sought to define observed differences in the radiographic appearance of implants relative to the joint line, in prone and supine positions.

**Methods:**

A 3.5 mm tubular plate and a 3.5 mm posterior distal tibial periarticular plate were applied sequentially to 3 individual cadaveric legs, via a posterolateral approach. The tubular plate was positioned to simulate buttress fixation and the posterolateral plate placed more distally. Each limb was secured on a custom jig and radiographs were taken on a mobile c-arm fluoroscopy machine with a calibration ball. A series of prone AP, supine PA and mortise radiographs were taken. Prone radiographs were also taken in different degrees of caudal tilt to simulate knee flexion which occurs in practice, during intraoperative positioning. Plate tip-joint line distances were measured and Mann–Whitney *U* tests performed.

**Results:**

There was no statistically significant difference in plate tip-joint line distance when comparing equivalent prone and supine views (PA/AP or mortise). However, significant differences in apparent implant position were noted with alterations in caudal tilt. When taking a prone image, when the knee is flexed to 20 degrees, the plate tip will appear 6.5–8.5 mm more proximal than in the equivalent supine image where the knee is extended and the fluoroscopy beam is orthogonal to the anatomic axis of the tibia.

**Conclusion:**

Observed differences in radiographic appearance of metalwork in the prone and supine position are most likely due to knee flexion and the resulting variation in the angle of the fluoroscopy beam, rather than projectional differences between supine and prone views. Surgeons should be alert to this when analysing intraoperative images.

## Introduction

The posterior malleolus of the distal tibia may be fractured in unstable ankle and pilon fractures; operative reduction and fixation is often indicated and this is commonly performed through a posterolateral approach which provides good access to the Volkmann fragment and any associated distal fibula fractures [1–3] – the leg may be positioned prone to optimise access. Once the fragment has been reduced and fixed, anteroposterior (AP), mortise and lateral radiographs are obtained to confirm plate position and fracture fixation. The complex fracture patterns seen in these injuries often require the leg to be repositioned supine to complete any necessary anterior or medial fixation, after which final posteroanterior (PA), mortise and lateral views are obtained.

With the patient prone the limb is supported by a bolster or pillow, which leads to knee flexion [1–3] and results in a variable angulation of the tibia relative to the vertical fluoroscopy beam. When the patient is supine the heel typically lies flat on the operating table. Operating in this manner allows optimal access for fracture fixation but can lead to different appearances of implant positioning on radiographic views. Radiographic studies of fixations at the distal radius, proximal humerus and medial malleolus have demonstrated that manipulating the angle and orientation of the image intensifier beam can dramatically change the surgeon’s assessment of implant position in relation to the joint line [4–7]. This study seeks to define the difference in apparent implant position between prone and supine limb positions during fixation of the posterior malleolus. No prior study in the English literature has described or investigated this.

## Materials and Methods

### Specimen Preparation

Three adult cadaveric lower extremity specimens (two male, left sided; one female, right sided) were used. Preliminary AP and lateral radiographs confirmed the absence of significant bony deformity, previous fractures or other osseous pathology.

A standard posterolateral approach was performed by the authors [2]. The incision was extended proximally to ensure visibility of the metaphyseal–diaphyseal junction and distally to ensure visibility of the joint line and posterior malleolus.

Two plates were selected for fixation: (i) a 3.5 mm precontoured posterior distal tibia periarticular plate (EVOS, Smith & Nephew, Watford, UK) and (ii) a 7-hole one-third tubular plate (EVOS, Smith & Nephew, Watford, UK). Plates were applied according to manufacturer’s instructions and fixed using 3.5 mm cortical screws. The posterolateral distal tibia plate was positioned in its natural resting position, close to the joint line. The one-third tubular plate was contoured and positioned more proximally to imitate buttress mode fixation of a Volkmann fragment; the position was standardised across specimens such that the distal tip of the plate was level with the most posterior prominence of the posterior malleolus. All plates were positioned superior to the joint line, confirmed by direct visualisation and lateral radiographs. Each cadaveric specimen had both plates applied sequentially.

**Fig. 1:**
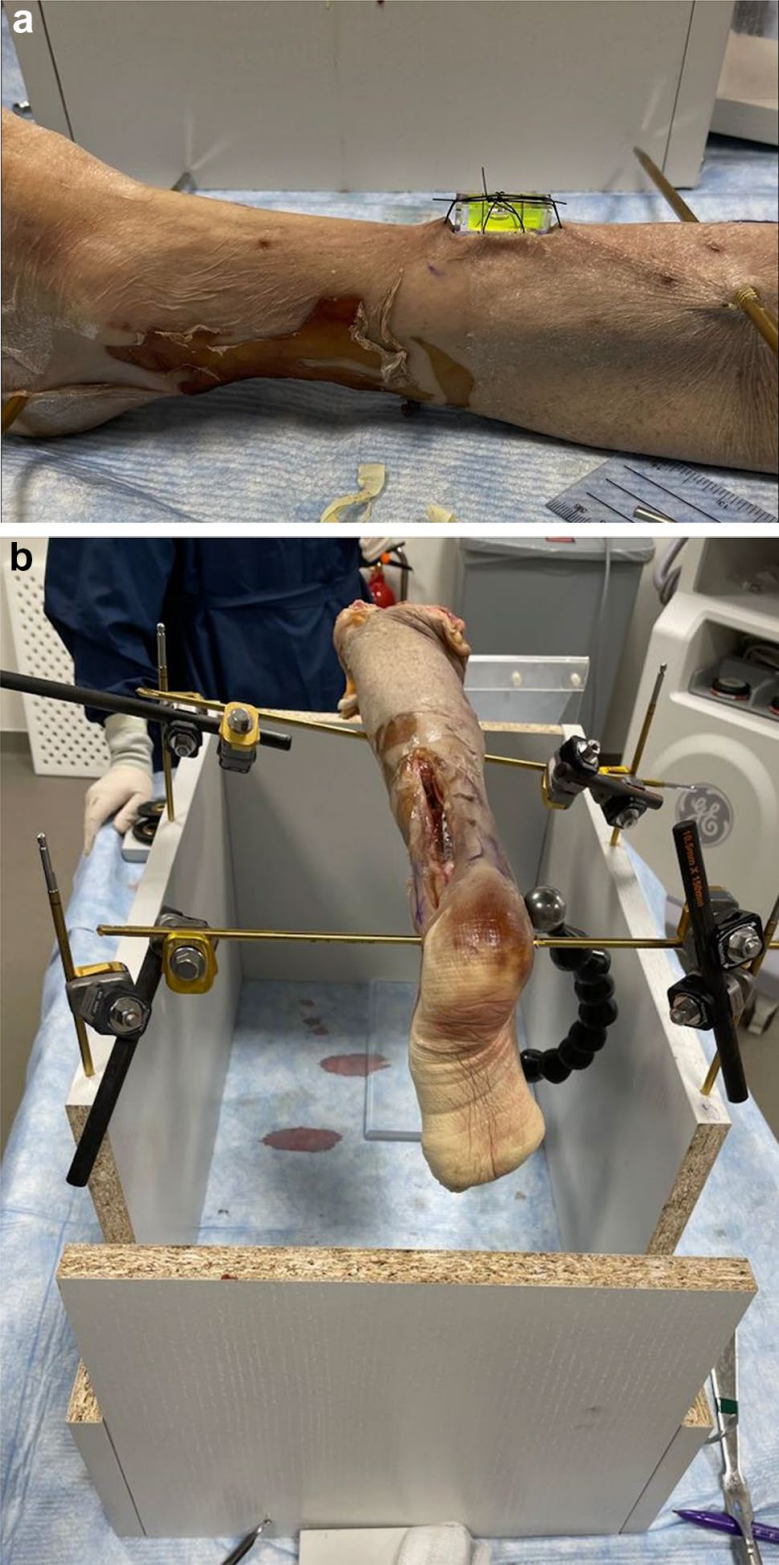
**a** Photograph of the spirit level sutured to the lower limb specimen to control sagittal plane angulation of the specimen relative to the c-arm. **b** Photograph of the specimen mounted onto custom built jig, with calibration ball positioned adjacent to the medial malleolus

After plate application each specimen was laid flat on the operating table and a spirit level was sutured overlying the tibial crest at a point where the bubble was central, such that the tibial crest was horizontal (Fig. 1a). The specimen was mounted onto a custom built positioning jig and held in place with external fixation pins, clamps and rods (Smith & Nephew, Watford, UK). The position was adjusted such that the spirit level bubble was again central (Fig. 1b).

### Obtaining Radiographs

Imaging was taken using a mobile c-arm fluoroscopy machine (GE Healthcare, Chicago, IL, USA), with the image intensifier positioned above the X-ray tube throughout, with the exception of the lateral views. A 25.4-mm-diameter calibration ball (OrthoMark, RolleSolutions Inc., Castro Valley, California, USA) was placed adjacent to the ankle, equidistant from the c-arm detector, to enable calibrated measurements to be taken.

A lateral image was taken to confirm appropriate plate positioning superior to the joint line. This was followed by supine PA and mortise views with the fluoroscopy beam vertical (0° caudal tilt), and a series of prone AP and mortise views with varying degrees of caudal tilt (0, 10, 15, 20°) to simulate the varying degrees of knee flexion that result from intraoperative limb positioning. The c-arm was manipulated with respect to the specimen as this was more practical and reproducible than the alternative method of tilting the specimen with respect to the c-arm.

A true mortise image was judged to be one in which the talar clear space medially, laterally and superiorly was equal, as agreed by the authors. As the standard radiographic method of taking a mortise view is to internally rotate the leg by 15° from the AP/PA position, a true AP/PA view was subsequently obtained by applying 15 degrees of right or left anterior oblique fluoroscopy beam angulation [8].

### Image Analysis

Images were uploaded to TraumaCad (Brainlab AG, Munich, Germany) software and calibrated by specifying the diameter of the calibration ball used. For each set of images the plate tip to joint line distance was measured independently in a standardised fashion by 3 observers (PP, ZL, PB).

**Fig. 2:**
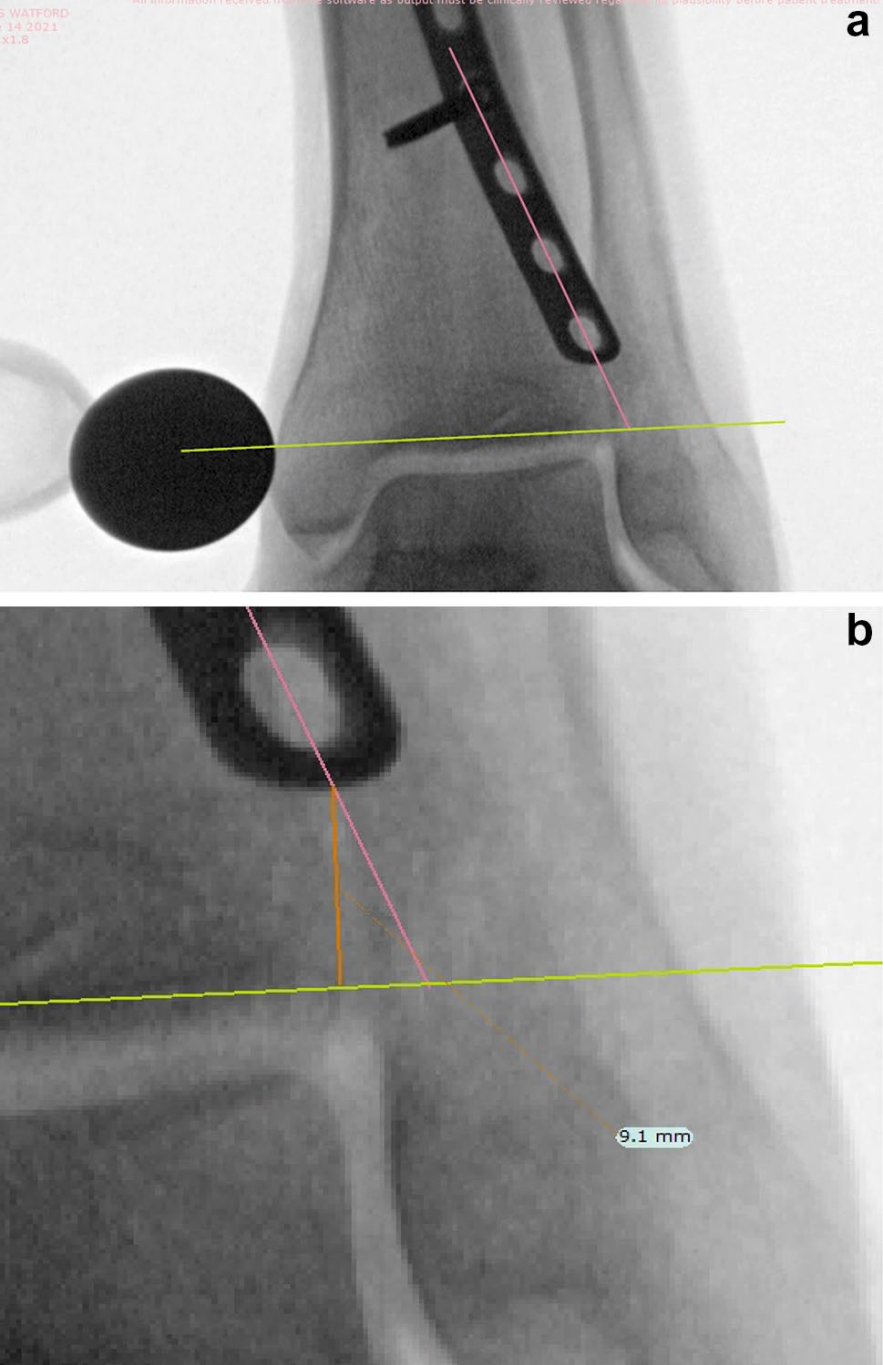
**a** and **b** Representative measurements on TraumaCad. The plate tip to joint line distance was calculated for both plates by drawing a transverse line (green line) through the joint line which was visually estimated as the thickest point of the articular surface of the distal tibia. Plate tip position was estimated by drawing a line through the centre of the holes corresponding to the midline of the tubular plate (pink line). The shortest distance between the two lines (orange line) was then measured

For PA, AP and mortise views this was the shortest distance from the midpoint of the distal end of the plate to the joint line (Fig. 2a and b). Data were analysed in Microsoft Excel (Microsoft Corporation, Redmond, Washington, USA). Mann–Whitney U tests with significance set to a *p* value < 0.05 were performed for statistical analysis.

## Results

10 plate-joint line distances were recorded by 3 independent observers for each of 6 plate–specimen combinations, giving a total of 180 measurements.

### Posterior Distal Tibia Precontoured Plate

#### Effect of Supine vs Prone Positioning on AP/PA and Mortise Views

**Fig. 3:**
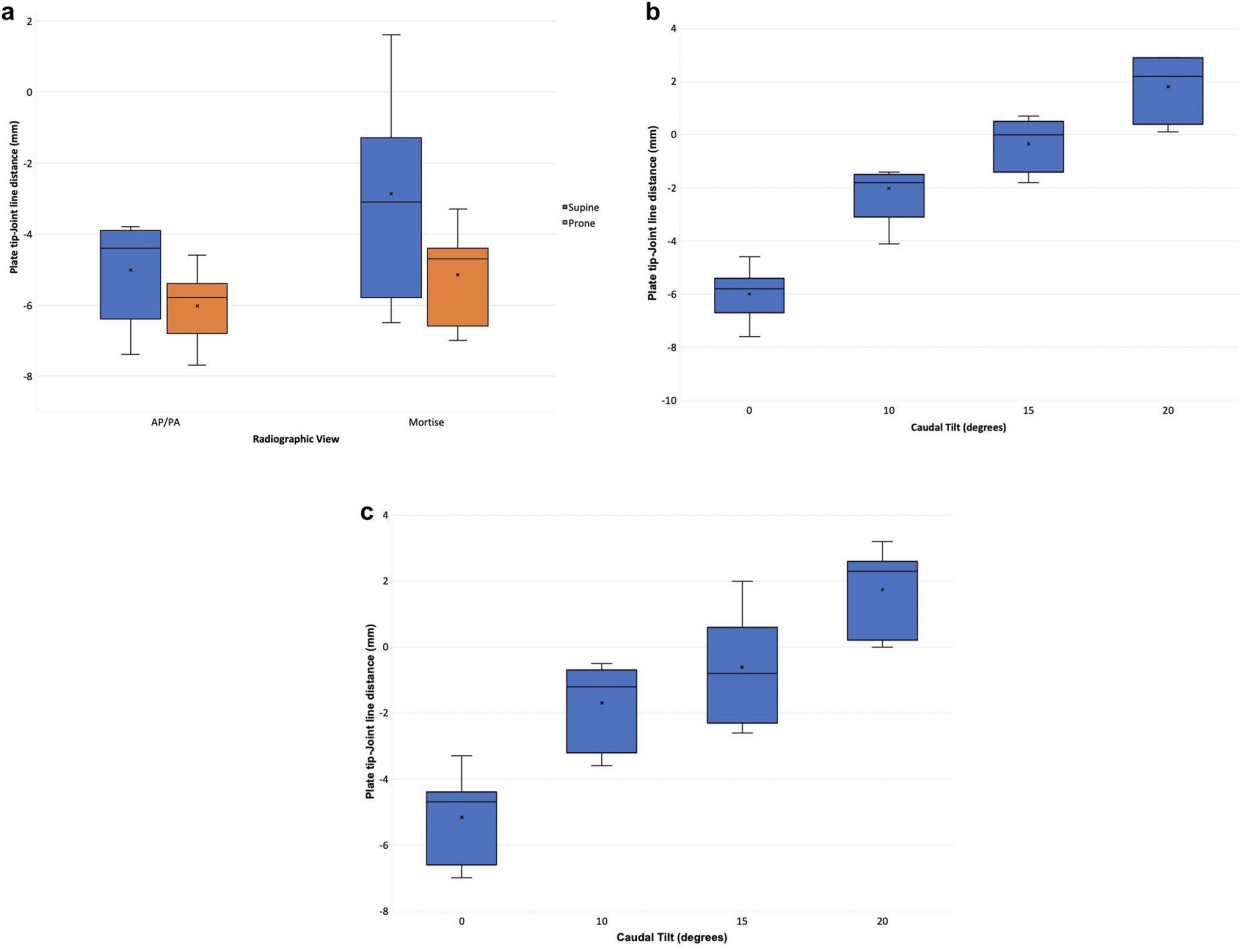
**a** Plate tip-joint line distances for the periarticular plate on AP/ PA and mortise views with 0° caudal tilt in supine and prone positions. **b** Plate tip-joint line distances for the periarticular plate on AP views in the prone position, showing the effect of increasing caudal tilt. **c** Plate tip-joint line distances for the periarticular plate on mortise views in the prone position, showing the effect of increasing caudal tilt

The plate tip appeared distal to the joint line on AP/PA and mortise views in both supine and prone positions with 0° caudal tilt, averaged across the 3 specimens (Fig. 3a). Prone positioning caused the plate tip to appear slightly more distal in comparison to the equivalent supine views, but this difference was not statistically significant.

On the AP/PA views, prone positioning caused the plate tip to appear an average of 1.0mm more distal compared to the equivalent supine views (difference = 1.1, 1.5, 0.3mm for specimens 1, 2, 3, respectively, *p* = 0.103).

On the mortise views, prone positioning caused the plate tip to appear an average of 2.3mm more distal compared to the equivalent supine views (difference = 5.0, 1.2, 0.7mm for specimens 1, 2, 3, respectively, *p* = 0.064).

#### Effect of Caudal Tilt on AP/PA Views

The plate tip appeared distal to the joint line on prone AP views with 0°, 10° and 15° caudal tilt at – 6.0 mm, – 2.0 mm and – 0.3 mm, respectively, averaged across specimens (Fig. 3b). The plate tip appeared proximal to the joint line at 20°caudal tilt, at a distance of 1.8 mm. The change in apparent plate tip position with each 5–10° increment to caudal tilt was statistically significant (0°–10° *p* < 0.001; 10°–15° *p* = 0.034; 15°–20° *p* = 0.014).

Beyond 10°, each additional 5° caudal tilt increased the plate-joint line distance by a mean of 1.9 mm (range 1.1–2.5 mm). The mean difference in the plate-joint line distance between the prone AP views with 0° vs 20° caudal tilt was 7.8 mm (range 7.4–8.5 mm).

#### Effect of Caudal Tilt on Mortise Views

The plate tip appeared distal to the joint line on prone mortise views with 0°, 10° and 15° caudal tilt at – 5.2 mm, – 1.7 mm and – 0.6 mm, respectively, averaged across specimens, and appeared an average of 1.7 mm proximal to the joint line at 20°caudal tilt (Fig. 3c). The change in apparent plate tip position with each increment to caudal tilt was statistically significant between 0° and 10° *p* < 0.001; 15° and 20° = 0.004 but not between 10° and 15° *p* = 0.10.

Beyond 10°, each additional 5° caudal tilt increased the plate-joint line distance by a mean of 1.7 mm (range 0.7–2.8 mm). The difference in the plate-joint line distance between the prone AP views with 0° vs 20° caudal tilt was 6.9 mm (range 6.5–7.3 mm).

### One-Third Tubular Plate

#### Effect of Supine vs Prone Positioning on AP/PA and Mortise Views

**Fig. 4:**
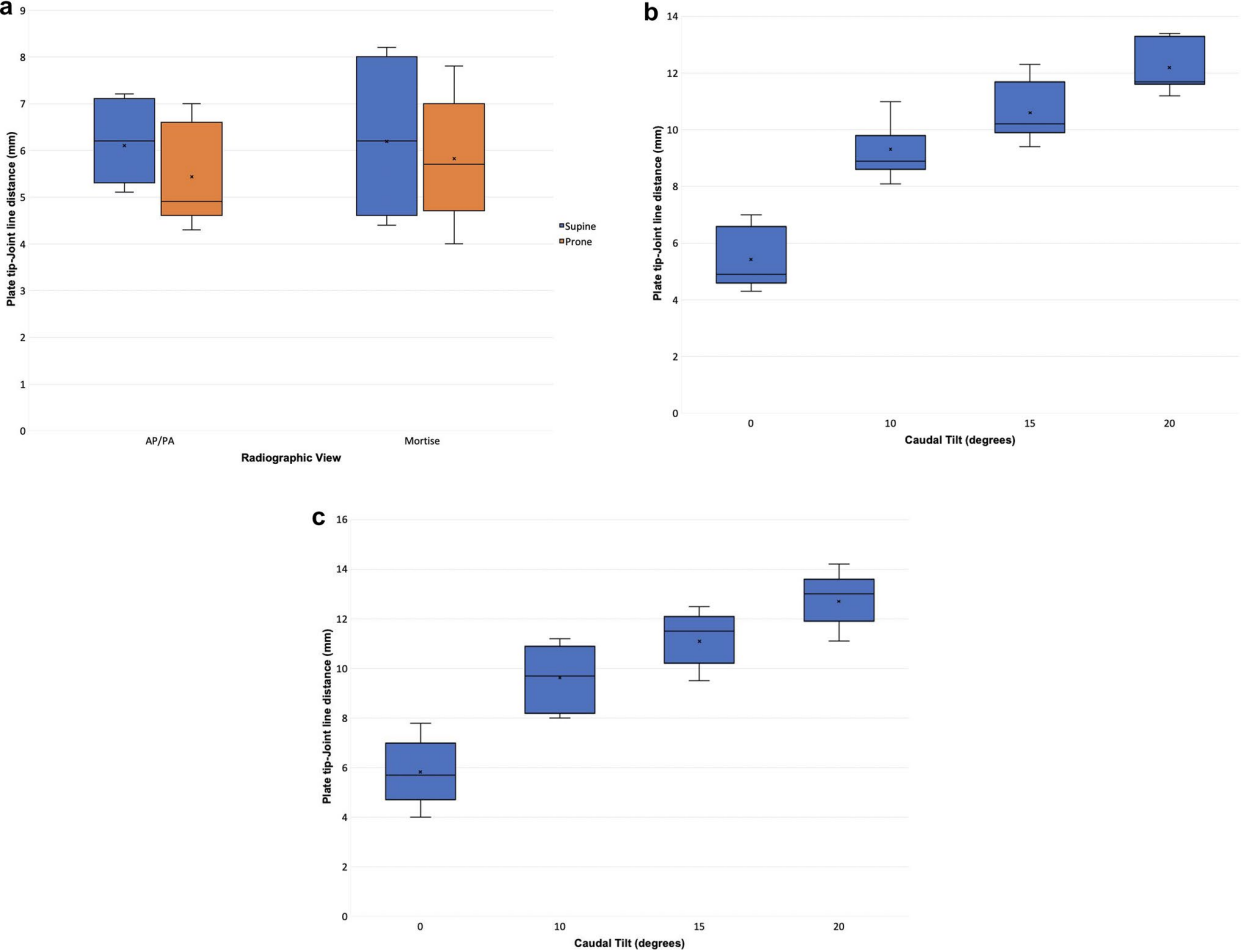
**a** Plate tip-joint line distances for the one-third tubular plate on AP/ PA and mortise views with 0° caudal tilt in supine and prone positions. **b** Plate tip-joint line distances for the one-third tubular plate on AP views in the prone position, showing the effect of increasing caudal tilt. **c** Plate tip-joint line distances for the one-third tubular plate on mortise views in the prone position, showing the effect of increasing caudal tilt

In all 3 specimens the plate tip appeared proximal to the joint line on AP/PA and mortise views in both supine and prone positions with 0° caudal tilt (Fig. 4a).

Prone positioning caused the plate tip to appear minimally more distal in comparison to the equivalent supine views; however, the observed differences were not statistically significant: on the PA/AP views, prone positioning caused the plate tip to appear more distal by an average of 0.7mm compared to the equivalent PA supine views (difference = 0.7, 1.0, 0.4mm for specimens 1, 2, 3, respectively, *p* = 0.056). On the mortise views, prone positioning also caused the plate tip to appear minimally more distal by an average of 0.4mm compared to the equivalent supine views (difference = 0.2, 0.3, 0.7mm for specimens 1, 2, 3, respectively, *p* = 0.268).

#### Effect of Caudal Tilt on AP/PA Views

The plate tip appeared proximal to the joint line on all prone AP views, measuring an average of 5.4 mm, 9.3 mm, 10.6 mm and 12.2 mm at caudal angles of 0°, 10°, 15° and 20°, respectively (Fig. 4b). The change in apparent plate tip position with each increment to caudal tilt was statistically significant (0°–10° *p* < 0.001; 10°–15° *p* = 0.011; 15°–20° = 0.014).

Beyond 10°, each additional 5° caudal tilt increased the plate-joint line distance by a mean of 1.4 mm (range 1.1–1.8 mm). The difference in the plate tip-joint line distance between the prone AP views with 0° vs 20° caudal tilt was 6.8 mm (range 6.6–7.0 mm).

#### Effect of Caudal Tilt on Mortise Views

The plate tip appeared proximal to the joint line on prone AP views, measuring 5.8 mm, 9.6 mm, 11.1 mm and 12.7 mm at caudal tilts of 0°, 10°, 15° and 20°, respectively, averaged across specimens (Fig. 4c). The change in apparent plate tip position with each increment to caudal tilt was statistically significant (0°–10° *p* =  < 0.001; 10°–15° *p* = 0.017; 15°–20° = 0.012).

Beyond 10°, each additional 5° caudal tilt increased the plate-joint line distance by a mean of 1.5 mm (range 1.2–1.7 mm). The difference in the plate-joint line distance between the prone AP views with 0° vs 20° caudal tilt was 6.9 mm (range 6.5–7.1 mm).

## Discussion

We have described the radiographic changes seen with alterations in limb positioning during posterior malleolus fixation – to our knowledge, this is this first study to investigate this phenomenon.

In all plates across all specimens, there was no statistically significant difference in plate tip-joint line distance when comparing equivalent prone and supine views (PA/AP or mortise). However, significant differences in apparent implant position were noted with alterations in caudal tilt. This suggests that observed clinical differences are most likely due to limb positioning with knee flexion and the resulting variation in the angle of the fluoroscopy beam relative to the distal tibial articular surface. When taking a prone image, when the knee is flexed to 20 degrees, the plate tip will appear 6.5–8.5 mm more proximal than in the equivalent image where the knee is extended and the fluoroscopy beam is orthogonal to the anatomic axis of the tibia.

Our results show that the issue of implants falsely appearing to be distal to the joint line did not arise with the more proximally positioned tubular plate, but did occur with the periarticular precontoured plate – likely because this implant is designed for more distal positioning. All periarticular plates were positioned under direct vision of the distal tibia articular surface, such that the tip was proximal to the distal margin of the posterior malleolus. However, 20° of caudal tilt was required before all plate tips appeared proximal to the joint line on AP/PA and mortise views.

This has implications for surgeons undertaking posterior malleolus fixation as they will need to carefully consider both the limb and c-arm position when interpreting images: surgeons should expect fixation constructs to appear relatively more superior during the initial phase of surgery, in which the limb is in prone position with the knee flexed and a bolster positioned beneath the limb, which confers a higher degree of caudal tilt. In the case of periarticular or very distally positioned plates, this difference may be sufficient to create the false appearance of intra-articular implants. This study may help surgeons understand and interpret their intraoperative radiographs during posterior malleolus fixation, and highlights the importance of the lateral view in combination with AP/PA and mortise views in confirming satisfactory implant position.

This study is limited by small sample size; however, we have developed a robust methodology which may be replicated in future laboratory or clinical studies to validate results. Resting alignment of the cadaveric limb on the custom jig was controlled using a spirit level – the authors acknowledge that this instrument may introduce a degree of subjectivity. We took further steps to limit error by standardising limb position and radiographic views and developed a standardised protocol for measurements which were taken independently by three observers.

## Conclusion

Differences noted intraoperatively in the plate tip-joint line distance on prone vs supine AP/PA/mortise views are most likely due to knee flexion induced by a supporting bolster (simulated in this study by caudal tilt of the c-arm), and the surgeon should be alert to this. This effect should be investigated further in a clinical study to enhance surgeons’ abilities to analyse fixations of the posterior malleolus.
